# La Grippe Again

**Published:** 1891-03

**Authors:** Wm. Steinrauf

**Affiliations:** St. Charles, Mo.


### LA GRIPPE AGAIN.

WM. STEINRAUF, M. D., ST. CHARLES, MO.

La grippe has again appeared in our city. Those escaping last winter are attacked this winter. The symptoms are somewhat changed this season. Sneezing is almost entirely absent. Headache is more in the form of a neuralgia, with terrible pains over the left eye. Backache is only of moderate intensity. Vomiting and diarrhœa predominate; especially the latter. There is no fever, but the heart's action is very labored. Dullness of the eyes, and in many cases there is much pain accompanying the diarrhœa.

The *Law*, as usual, has again proved itself all-sufficient. Whilst our allopathic friends have advised Castor Oil with Laudanum, Calomel, and Quinine, we gave, according to the indications, Gelsemium, Bryonia, Lac-caninum, and Belladonna. They all recovered in a very short time. Where the disease had been suppressed last winter to reappear now, Pulsatilla cured. Lac-caninum was used more than any other remedy.

P. S.—Since writing the above, I have had some experience with a nosode. Catarrhus-intestinalus$^{cm}$ was given in some twenty or more cases in the very beginning of the diarrhœa, and the results were indeed marvellous. I used this nosode only as an experiment, there being no provings of it, and only in the beginning of the bowel trouble.

Lac-caninum was much used, and where indicated proved to be a grand remedy.

### AN EXPLANATION.

I am very thankful to Dr. Griffith, of Edina, Mo., for calling my attention to a seeming inconsistency on my part in relating a case of erysipelas in the last December issue of THE HOMŒOPATHIC PHYSICIAN.

With the cure of the erysipelas in my seventy-seven-year-old patient, I spoke of having at the same time cured him of a chronic eczema of the nose, which had existed over twenty years. This happened. In the article referred to I said the patient was cured of his erysipelas during la grippe period last winter, and that two years later there was no recurrence of the eczema. Here is a seeming contradiction. I was writing an article on chronic diarrhœa for one of our journals at the time, and two years later the patient reported himself well, and I thus got cases and dates mixed. Hence the mistake. It is about a year ago since the erysipelas patient got well of his erysipelas, and when I passed his place of business to-day and inquired after his eczema, he triumphantly replied: "Gone, never to return." Let us hope so.

